# Long-Term Safety and Immunogenicity of AZD1222 (ChAdOx1 nCoV-19): 2-Year Follow-Up from a Phase 3 Study

**DOI:** 10.3390/vaccines12080883

**Published:** 2024-08-03

**Authors:** Kathryn Shoemaker, Karina Soboleva, Angela Branche, Shivanjali Shankaran, Deborah A. Theodore, Muhammad Bari, Victor Ezeh, Justin Green, Elizabeth Kelly, Dongmei Lan, Urban Olsson, Senthilkumar Saminathan, Nirmal Kumar Shankar, Berta Villegas, Tonya Villafana, Ann R. Falsey, Magdalena E. Sobieszczyk

**Affiliations:** 1 Biometrics, Vaccines & Immune Therapies, BioPharmaceuticals R&D, AstraZeneca, Gaithersburg, MD 20878, USA; kathryn.shoemaker@astrazeneca.com (K.S.); dongmei.lan@astrazeneca.com (D.L.); 2 Clinical Development, Vaccines & Immune Therapies, BioPharmaceuticals R&D, AstraZeneca, Gaithersburg, MD 20878, USA; karina.soboleva1@astrazeneca.com (K.S.); victor.ezeh@astrazeneca.com (V.E.);; 3 Division of Infectious Diseases, Department of Medicine, University of Rochester, Rochester, NY 14627, USA; angela_branche@urmc.rochester.edu; 4 Division of Infectious Diseases, Rush University Medical Center, Chicago, IL 60612, USA; shivanjali_shankaran@rush.edu; 5 Division of Infectious Diseases, Department of Medicine, Vagelos College of Physicians and Surgeons, New York-Presbyterian/Columbia University Irving Medical Center, New York, NY 10032, USA; dat2132@cumc.columbia.edu (D.A.T.);; 6 Formerly Patient Safety, Vaccines & Immune Therapies, BioPharmaceuticals R&D, AstraZeneca, Cambridge CB2 0AA, UK; muhammad.bari@hotmail.com; 7 Clinical Development, Vaccines & Immune Therapies, BioPharmaceuticals R&D, AstraZeneca, Cambridge CB2 0AA, UK; 8 Formerly Translational Medicine, Vaccines & Immune Therapies, BioPharmaceuticals R&D, AstraZeneca, Gaithersburg, MD 20878, USA; beth.j.kelly@gmail.com; 9 Clinical Development, Vaccines & Immune Therapies, BioPharmaceuticals R&D, AstraZeneca, 431 83 Gothenburg, Sweden; urban.x.olsson@astrazeneca.com; 10 Patient Safety, Chief Medical Office, R&D, AstraZeneca, Bangalore 560045, India; senthilkumar.saminathan@astrazeneca.com (S.S.); nirmalkumar.shankar@astrazeneca.com (N.K.S.); 11 Clinical Operations, Vaccines & Immune Therapies, BioPharmaceuticals R&D, AstraZeneca, Mississauga, ON L4Y 1M4, Canada; berta.villegas@astrazeneca.com; 12 Department of Medicine, Infectious Diseases, University of Rochester School of Medicine and Dentistry, Rochester, New York, NY 14642, USA; ann_falsey@urmc.rochester.edu; 13 Infectious Disease, Rochester Regional Health, Rochester, New York, NY 14617, USA

**Keywords:** AZD1222 (ChAdOx1 nCoV-19), COVID-19, long-term safety, SARS-CoV-2, humoral immunogenicity, COVID-19 vaccine, Omicron variant

## Abstract

A better understanding of the long-term safety, efficacy, and immunogenicity of COVID-19 vaccines is needed. This phase 3, randomized, placebo-controlled study for AZD1222 (ChAdOx1 nCoV-19) primary-series vaccination enrolled 32,450 participants in the USA, Chile, and Peru between August 2020 and January 2021 (NCT04516746). Endpoints included the 2-year follow-up assessment of safety, efficacy, and immunogenicity. After 2 years, no emergent safety signals were observed for AZD1222, and no cases of thrombotic thrombocytopenia syndrome were reported. The assessment of anti-SARS-CoV-2 nucleocapsid antibody titers confirmed the durability of AZD1222 efficacy for up to 6 months, after which infection rates in the AZD1222 group increased over time. Despite this, all-cause and COVID-19-related mortality remained low through the study end, potentially reflecting the post-Omicron decoupling of SARS-CoV-2 infection rates and severe COVID-19 outcomes. Geometric mean titers were elevated for anti-SARS-CoV-2 neutralizing antibodies at the 1-year study visit and the anti-spike antibodies were elevated at year 2, providing further evidence of increasing SARS-CoV-2 infections over long-term follow-up. Overall, this 2-year follow-up of the AZD1222 phase 3 study confirms that the long-term safety profile remains consistent with previous findings and supports the continued need for COVID-19 booster vaccinations due to waning efficacy and humoral immunity.

## 1. Introduction

Vaccines have been vital in providing protection against the coronavirus disease 2019 (COVID-19) pandemic. Over 50 COVID-19 vaccines have been authorized for use worldwide, with the first doses being administered in late 2020 and early 2021 [1,2,3,4]. Throughout their first year of deployment, COVID-19 vaccines are estimated to have saved 19.8 million lives [5]. AZD1222 (ChAdOx1 nCoV-19) has been among the most widely used of the COVID-19 vaccines, with more than 3 billion doses distributed in over 170 countries [6,7]. Although the World Health Organization (WHO) has now declared an end to the acute phase of the COVID-19 public health emergency, vaccination remains a critical strategy for providing ongoing protection in individuals vulnerable to severe disease [8]. Consequently, the WHO recommends the continued use of booster dosing strategies to maintain immunity against severe acute respiratory syndrome coronavirus 2 (SARS-CoV-2), especially in the elderly, immunocompromised, and those with high-risk comorbidities [8].

Although vaccines such as AZD1222, developed against the original, ancestral SARS-CoV-2 variant, were studied extensively prior to authorization by regulatory authorities, their widespread use to mitigate the global burden of COVID-19 highlights a need to provide extended clinical safety data [8,9,10,11]. Further analysis of the long-term efficacy and immunogenicity for ancestral SARS-CoV-2 COVID-19 vaccines may also add to our understanding of the durability of protection conferred against severe disease and death from more recent SARS-CoV-2 variants [12,13,14]. Additionally, extended clinical data for AZD1222 are important as the ChAdOx1 vaccine platform used for AZD1222 remains an active area of scientific collaboration and discovery, particularly in global efforts to prepare for future pandemics [15].

This phase 3, randomized, placebo-controlled study, which evaluated a two-dose primary series of AZD1222 (NCT04516746), enrolled a diverse population in the USA, Chile, and Peru between August 2020 and January 2021 and provides key clinical data on AZD1222. The primary analysis, reported when primary endpoint events were met after a median of 2-months follow-up, demonstrated that AZD1222 was well tolerated, immunogenic, and was associated with a 74.0% vaccine efficacy (VE) against symptomatic COVID-19 [11]. A 6-month interim analysis from the double-blind phase of the study noted that no emergent safety issues were detected in the AZD1222 group and that protection was durable through 6 months of follow-up. VE against SARS-CoV-2 infection was 67.4%, while VEs against symptomatic and severe COVID-19 were 65.1% and 92.1%, respectively [16]. Clinical immunogenicity assessments have provided insight into the potential immune dynamics underlying the protection afforded by AZD1222. Robust humoral immunogenicity, including both anti-SARS-CoV-2 spike antibodies (Abs) and neutralizing Abs (nAbs), as well as mucosal immunogenicity, has been observed post-AZD1222 across adults (≥18 years) of all ages [16,17]. Additionally, following breakthrough infection, the humoral, cellular, and mucosal immunogenicity afforded by AZD1222 vaccination was inversely correlated with SARS-CoV-2 virologic outcomes and was shown to attenuate SARS-CoV-2 viral loads and shorten the duration of COVID-19 symptoms [17,18]. 

As the COVID-19 pandemic has evolved, so too has the real-world context of this study. Since 2020, four, seven, and four monovalent COVID-19 vaccines have been approved or granted Emergency Use Authorization (EUA) in the USA, Chile, and Peru, respectively; AZD1222 was approved for use in Chile and Peru [4]. When participants became eligible to receive a licensed or authorized COVID-19 vaccine, they were unblinded to intervention assignment: over the study duration, this occurred for the majority of participants. Moreover, throughout the course of the study, waves of infection continued in the regions of enrollment, driven by the emergence of SARS-CoV-2 variants [19]. For example, the beginning of the study was characterized by a high prevalence of Alpha and Gamma variants; however, from mid-2021, there was a rapid spread of the Delta variant across all study locations, followed by Omicron, which quickly became the dominant variant in circulation [20,21]. Consequently, boosting strategies were widely recommended to counter waning primary-series immunity [8]. Despite these additional mitigation efforts, successive waves of Omicron subvariants with enhanced transmissibility and immune evasion have continued to be the predominant drivers of global COVID-19 cases [20,22,23]. 

We sought to evaluate the long-term safety of AZD1222, including after boosting, from initiation to completion of the phase 3 study, a period spanning a full 2 years of follow-up. We also sought to understand the durability of protection and immunogenicity afforded by a two-dose AZD1222 primary series within the context of endemic SARS-CoV-2 circulation.

## 2. Materials and Methods

### 2.1. Study Design and Participants

This phase 3, randomized, placebo-controlled study for AZD1222 (ClinicalTrials.gov Identifier: NCT04516746) was conducted across 88 sites in the USA, Chile, and Peru. Full details of the study design and participants have been reported previously [11,16]. In brief, the study enrolled participants aged ≥18 years who were healthy or had medically stable chronic diseases and who were at increased risk of COVID-19. Participants were excluded if they had a history of laboratory-confirmed SARS-CoV-2 infection, any confirmed or suspected immunosuppressive or immunodeficient state, recurrent severe infections, or the use of immunosuppressant medication (except for participants living with well-controlled HIV on stable antiretroviral therapy). The full eligibility criteria are available in the protocol published previously [11,16].

This study was conducted in compliance with the principles of the Declaration of Helsinki and the International Council for Harmonization Good Clinical Practice guidelines. The protocol and amendments for this study were approved by the ethics committee or institutional review board at each center, and all participants provided informed consent prior to enrollment.

### 2.2. Treatment, Randomization, and Masking

Participants were randomized 2:1 to receive either AZD1222 (5 × 10^10^ viral particles) or saline placebo administered via intramuscular injection on days 1 and 29 (4 weeks apart; −3/+7 days). Randomization was stratified by age (18–64 vs. ≥65 years), with a target of having 25% of the study population in the ≥65 years subgroup. Participants, investigators, and sponsor staff members involved in the study were blinded to randomization. For ethical reasons, participants could be unblinded for safety, for example, in an emergency when knowledge of the specific blinded study medication would affect the immediate management of the participants’ condition. Importantly, participants were also unblinded once they became eligible to receive a licensed or authorized COVID-19 vaccine outside of the study to facilitate the ability of participants to receive EUA vaccines. Further details of participant randomization have been previously published [11,16]. 

### 2.3. Procedures

Unsolicited adverse events (AEs) were recorded up to day 57 (28 days after each dose) at study visits and via telephone. Serious adverse events (SAEs), medically attended adverse events (MAAEs), and adverse events of special interest (AESIs) were recorded at study visits and via telephone up to day 730, regardless of unblinding or receipt of non-study COVID-19 vaccination. 

Participants self-monitored for COVID-19 qualifying symptoms up to day 360 and were prompted weekly by study sites; those with one or more qualifying symptoms attended an illness visit for the collection of nasopharyngeal swabs for SARS-CoV-2 reverse transcription polymerase chain reaction (RT-PCR) testing, as previously described [16]. Those participants with RT-PCR-confirmed SARS-CoV-2 infections continued to attend scheduled illness visits for up to 28 days, and nasopharyngeal swabs and saliva samples were sequenced to identify SARS-CoV-2 variants. All COVID-19 cases were therefore adjudicated up to day 360. After day 360, participants were asked to report COVID-19 illnesses, which were recorded as protocol-specified AESIs, but illness evaluation and sample collection were not performed. In addition, at the final study visit on day 730, participants were queried about COVID-19 illnesses that had not already been reported.

To assess the efficacy of the vaccine regardless of symptom presence or severity, serum samples were collected from participants at scheduled study visits on days 1, 29, 57, 90, 180, 360, and 730 (prior to dosing on days 1 and 29) for assessment of anti-SARS-CoV-2 nucleocapsid Abs. To evaluate the immunogenicity of AZD1222, the trial included a substudy comprising the first 3000 participants randomized in each age group in the USA: 1500 participants aged 18–55 years, 750 participants aged 56–69 years, and 750 participants aged ≥70 years. Participants in the substudy attended two additional study visits on days 15 and 43, with serum bring collected for further serological assessments at these visits. Serological assessments included pseudovirus neutralization assays for nAbs up to day 360 (not including day 90) and multiplex immunoglobulin G assays for anti-spike Abs up to day 730, both conducted against ancestral SARS-CoV-2. Procedures and testing were carried out as previously described (Supplementary Methods) [11,16,24].

### 2.4. Outcomes

The primary endpoints included reactogenicity (reported in full in the primary analysis), safety and tolerability, and efficacy. The primary safety endpoints included the incidence of non-serious AEs for 28 days after each dose (up to day 57), which have been reported previously [11,16], and long-term safety including SAEs, MAAEs, and AESIs from the time of signed informed consent up to day 730, which are presented herein.

The primary efficacy endpoint, the first occurrence of SARS-CoV-2 symptomatic illness as confirmed by a positive RT-PCR test and onset 15 days or more after the second vaccine dose in baseline seronegative participants, has been previously reported in full [11,16]. Exploratory endpoints assessing the durability of protection presented herein include time to SARS-CoV-2 infection regardless of symptoms or severity, measured as the first serologic response (negative at baseline and positive after baseline) for anti-SARS-CoV-2 nucleocapsid Abs from day 44 (≥15 days post-second dose), as well as the all-cause mortality and COVID-19-related mortality from day 1 to day 730.

Long-term humoral immunogenicity was assessed as a secondary endpoint through analysis of anti-spike Ab and nAb titers against ancestral SARS-CoV-2 from baseline to day 57 (28 days post-second dose) and at subsequent scheduled follow-up study visits. nAb titers were assessed up to the scheduled 1-year follow-up visit at study day 360. Anti-spike Ab levels were analyzed up to the scheduled 2-year follow-up visit at study day 730. 

### 2.5. Analysis Populations

Extended safety was examined in a safety population that included participants who received at least one dose of either AZD1222 or placebo and analyzed according to the intervention received. The receipt of a non-study COVID-19 vaccination was treated as an intercurrent event. Therefore, safety analyses are summarized for both periods prior to and after the receipt of non-study COVID-19 vaccination. 

Exploratory efficacy analyses included assessment in two populations. The incidence of the first positive response for anti-SARS-CoV-2 nucleocapsid Abs was assessed in the fully vaccinated analysis set (FVAS), which included all participants who were SARS-CoV-2 seronegative at baseline, received both doses, and remained in the study for ≥15 days after the second dose without a prior confirmed SARS-CoV-2 RT-PCR-positive infection. Additionally, the cumulative incidence of all-cause mortality and COVID-19-related mortality from day 1 to day 730 were assessed in a subset of baseline seronegative participants in the full analysis set (FAS), which included all participants who were randomized and received at least one dose of study intervention. All efficacy analyses were censored at the date of non-study COVID-19 vaccination or the date of last study contact, whichever occurred first.

Assessments of AZD1222 immunogenicity were performed in the immunogenicity substudy population, which included all substudy participants who received at least one dose of AZD1222 or placebo and who had no exclusionary protocol deviations with the potential to interfere with immunogenicity analyses. For timepoints after dosing (i.e., post-study day 29), the immunogenicity substudy population included participants who had received two doses of AZD1222 and had remained in the study for at least 15 days following completion of dosing. Baseline immunogenicity measurements were taken prior to the first dose of study intervention. Immunogenicity analyses were censored at the date of non-study COVID-19 vaccination, or the date of last study contact, whichever occurred first. 

### 2.6. Statistics

The censoring implications for analyses of the population for the period up to non-study COVID-19 vaccination are fully detailed by Sobieszczyk et al. [16]. Briefly, at the 6-month follow-up, 3518 (18.0%) and 6742 (76.0%) participants in the AZD1222 and placebo groups, respectively, had received non-study COVID-19 vaccinations [16]. Because all efficacy and immunogenicity analyses reported herein were censored at non-study COVID-19 vaccination, follow-up times were shorter in the placebo group compared with the AZD1222 group, prohibiting formal statistical comparison between groups. Booster vaccinations, defined as any COVID-19 vaccine dose (including AZD1222) received after a completed primary course, were not offered as part of this study and were therefore censored as non-study COVID-19 vaccinations. The wide availability of boosters during the 2-year follow-up period further limited the population for analyses censored at non-study COVID-19 vaccination [25,26]. To ensure a meaningful evaluation of the results, analyses were suspended at the point at which any group had <10% of participants remaining without a non-study COVID-19 vaccination. 

Frequencies of AEs are summarized descriptively, and no statistical analyses were planned for comparisons between groups. Due to the censoring implications described above, efficacy analyses are presented descriptively, without the calculation of vaccine efficacy, as planned. Immunogenicity analyses are reported descriptively as geometric mean titers (GMTs), as well as the first and third quartiles, interquartile range (IQR), and median values.

## 3. Results

### 3.1. Participants

**Figure 1:**
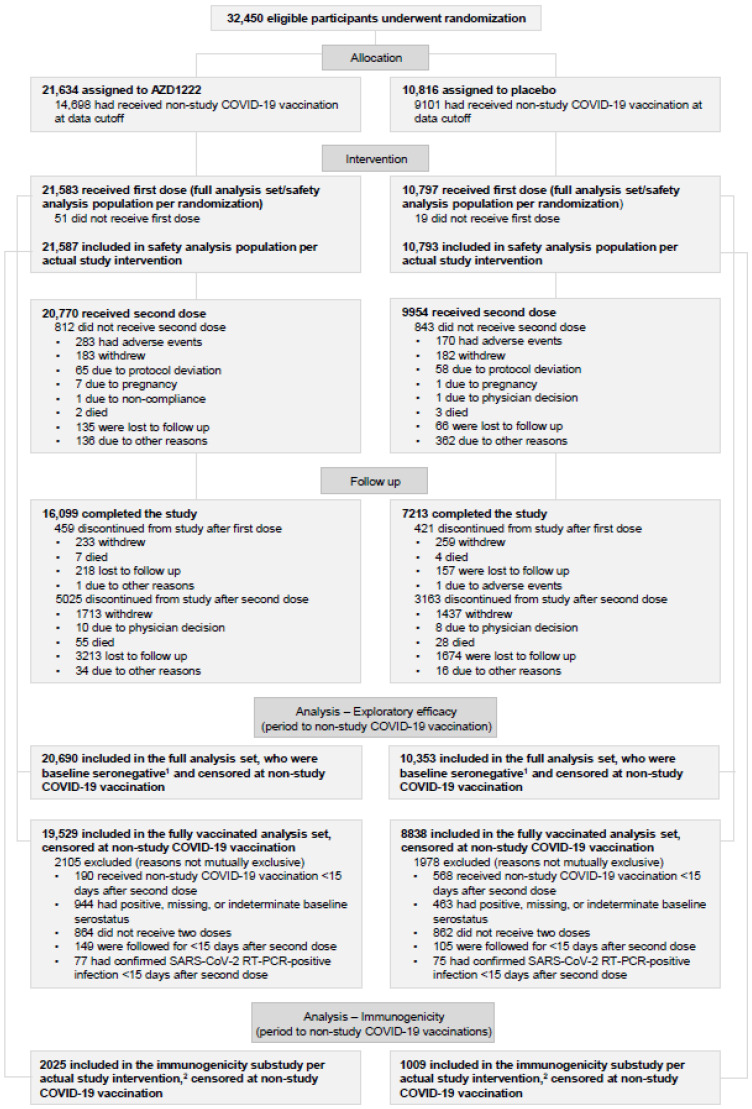
Participant disposition. ^1^ Serostatus at baseline was defined by the nucleocapsid Ab level as measured by the Roche Elecsys^®^ anti-SARS-CoV-2 nucleocapsid serology test (Covance CLS, Indianapolis, IN, USA). ^2^ Overall, 3042 (AZD1222: 2030; placebo: 1012) participants were enrolled into the substudy [16]. Substudy participants who received at least one dose of AZD1222 or placebo, and who had no exclusionary protocol deviations with the potential to interfere with immunogenicity analyses, were included in the immunogenicity substudy. Ab, antibody; COVID-19, coronavirus disease 19; RT-PCR, reverse transcription polymerase chain reaction; SARS-CoV-2, severe acute respiratory syndrome coronavirus 2.

The data cutoff for these analyses was 21 March 2023, covering a follow-up duration of 2 years (study day 730). As described previously, a total of 32,450 participants were enrolled between 28 August 2020 and 25 January 2021 and randomized (2:1) to receive AZD1222 (n = 21,634) or placebo (n = 10,816; Figure 1) [16]. 

Overall, 71.8% (AZD1222, 74.4% [n = 16,099]; placebo, 66.7% [n = 7213]) of the randomized participants completed the study despite a very high rate of unblinding (90.8% [n = 29,478]) due to participants becoming eligible to receive licensed or authorized COVID-19 vaccines. Participants receiving placebo were more likely to discontinue the study after unblinding (25.7%; n = 2783) than those receiving AZD1222 (19.0%; n = 4110). Among participants who discontinued the study, the most common reasons for discontinuation were loss to follow-up (AZD1222, 62.6% [n = 3431]; placebo, 51.1% [n = 1831]) and withdrawal (AZD1222, 35.5% [n = 1946]; placebo 47.3% [n = 1696]); one participant in the placebo group withdrew from the study before the second dose due to an AE of asphyxia, which resulted in death. 

By the end of the follow-up, most randomized participants in both groups had received a non-study COVID 19 vaccination (73.3%; n = 23,799) and were thus censored at the date of non-study COVID-19 vaccination for several analyses; however, the timing of censoring and reason for obtaining a non-study vaccination varied between groups. As would be expected, more placebo-treated participants received a non-study COVID-19 vaccination than AZD1222-treated participants (84.1% [n = 9101] vs. 67.9% [n = 14,698]), and more discontinued the study after receiving a non-study COVID-19 vaccine (20.0% [n = 2166] vs. 7.6% [n = 1648]). Booster vaccinations were not offered as part of the study and were treated as non-study COVID-19 vaccinations. As boosters became widely available during the extended follow-up period, 67.8% (n = 14,665) of participants in the AZD1222 group received a non-study COVID-19 booster vaccination.

Non-study COVID-19 vaccination, therefore, occurred at higher and faster rates in participants receiving placebo compared with participants receiving AZD1222. This resulted in shorter follow-up times in the placebo group for analyses censored at non-study COVID-19 vaccination (Table S1), prohibiting formal statistical comparison between groups. By day 240, less than 10% of placebo participants in the safety population who remained in the study had not received a non-study vaccination.

There were no changes from the 6-month interim analysis to the safety population or the FAS (Figure 1) [16]. The FVAS for the period up to non-study COVID-19 vaccination comprised 19,529 participants in the AZD1222 arm and 8838 participants in the placebo arm. The reasons for exclusion from this analysis population are detailed in Figure 1. The immunogenicity substudy comprised 2025 participants in the AZD1222 arm and 1009 participants in the placebo arm. Participant characteristics for the analysis populations are reported in Table S2. In the safety population, the majority of participants were male (55.6% [n = 18,013]), White (79.0% [n = 25,585]) and had at least one comorbidity at baseline (60% [n = 19,437]); the median age was 51.0 years (range: 18–100 years). Participant characteristics were well balanced across the analysis populations, and changes were minimal at this updated analysis compared with the 6-month and primary data [16].

### 3.2. Long-Term Safety

In the safety population, median follow-up duration after the first dose was 714.0 days (range: 1–864) for the AZD1222 group and 710.0 days (range: 1–839) for the placebo group (Table S1). For analyses censored at non-study COVID-19 vaccination, median follow-up duration after the first dose was considerably shorter for both groups: 328.0 days (range: 1–848) for the AZD1222 group and 100.0 days (range: 1–789) for the placebo group. Consequently, only 17.2% (n = 3718) and 3.1% (n = 335) of participants had follow-up data available for the full 2 years (study day 720) in the AZD1222 and placebo groups, respectively.

At 2-year follow-up, no emergent or unexpected safety signals were observed for AZD1222 (Table 1); the safety data for the placebo group are included in Table S3. In the period prior to non-study COVID-19 vaccination, 42 (0.2%) participants had AEs with an outcome of death and 43 (0.2%) had AEs leading to study discontinuation; none of these events were considered related to AZD1222. Causes of death not related to AZD1222 included cardiac disorders, respiratory disorders, infections, injury, neoplasms, pancreatic failure, dementia, suicide, and deep-vein thrombosis. SAEs occurred in 621 participants (2.9%), with SAEs being considered related to AZD1222 in 7 participants (<0.1%); AZD1222-related SAEs were myocardial infarction, retinal vein occlusion, mesenteric vein thrombosis, portal vein thrombosis, cerebral venous thrombosis, chronic inflammatory demyelinating polyradiculoneuropathy, hypoesthesia, paresthesia, and spontaneous abortion. MAAEs were reported in 4750 participants (22.0%), with 107 participants (0.5%) having MAAEs considered to be related to AZD1222. A total of 2516 participants (11.7%) reported AESIs, with 68 (0.3%) being considered as related to AZD1222; the majority of AESIs were participants reporting cases of COVID-19 (10%; n = 2151), which was registered as a pre-specified AESI for this study. 

Extremely rare events of thrombotic thrombocytopenia syndrome (TTS), also known as vaccine-induced immune thrombotic thrombocytopenia, have been detected after COVID-19 vaccination through post-trial pharmacovigilance [27,28]. No events of TTS were reported for the duration of this study. Prior to non-study COVID-19 vaccination, the incidence of participants reporting other specific AESIs, including thrombocytopenia (<0.1% [n = 5] vs. 0% [n = 0]), immune-mediated thrombocytopenia (<0.1% [n = 2] vs. <0.1% [n = 1]), deep-vein thrombosis (0.1% [n = 25] vs. <0.1% [n = 3]), cerebral venous thrombosis (<0.1% [n = 1] vs. <0.1% [n = 1]), thrombosis (<0.1% [n = 3] vs. 0% [n = 0]), pulmonary embolism (<0.1% [n = 21] vs. <0.1 [n = 1]), and Guillain–Barré syndrome (<0.1% [n = 1] vs. 0% [n = 0]), were similar in the AZD1222 and placebo groups, respectively.

Rates of AEs across categories were generally consistent between the period up to non-study COVID-vaccination and the period after non-study COVID-19 vaccination, with a slight increase in rates of AESIs after non-study COVID-19 doses (exposure-adjusted rates: 0.12 vs. 0.26). Rates of SAEs remained low (0.03), and no emergent safety issues were noted in participants who received non-study COVID-19 vaccination. 

### 3.3. Exploratory Efficacy

The durability of efficacy of primary-series AZD1222 was most apparent in the short-term period, from study day 44 (15 days post-second dose) to study day 180 (6 months post-first dose). In this period, the incidence of SARS-CoV-2 infection, measured as the rate of anti-SARS-CoV-2 nucleocapsid antibody seroconversion, was numerically lower in the AZD1222 group compared with the placebo group (66.72 vs. 217.15 per 1000 person-years; Table 2 and Table S4). Beyond 6 months, SARS-CoV-2 infection rates in the AZD1222 group increased over time, and after 1 year, 24.5% of the population (n/N = 1678/6850) had evidence of SARS-CoV-2 infection. Formal statistical comparisons with the placebo group were prohibited by the aforementioned differences in follow-up duration. 

**Figure 2:**
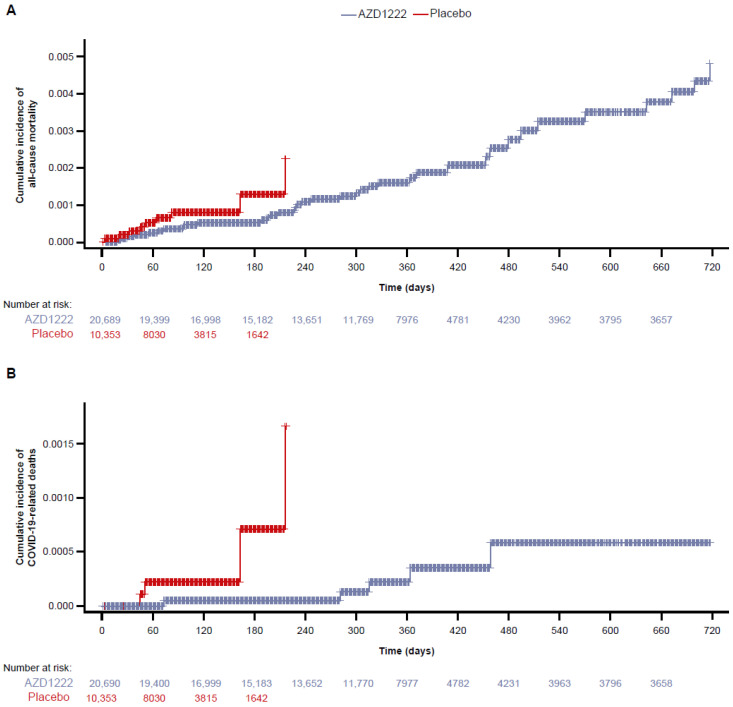
(**A**) All-cause mortality and (**B**) COVID-19-related mortality occurring through the study duration. Cumulative incidence of all-cause mortality and COVID-19-related mortality were assessed in the FAS population who were seronegative at baseline from day 1 to day 730. Participants were censored at non-study COVID-19 vaccination. The FAS included all randomized participants who received at least one dose of AZD1222 or placebo. The time to mortality event was measured from the time of first dose administration, and was calculated as: (date of event) − (date of first dose of AZD1222 or placebo) + 1. For censored participants, the censoring time was the time from the date of first dose of study intervention to the last observed time during the analysis period or licensed COVID-19 vaccine administration. Cumulative incidence curves were truncated at the point, at which <10% of participants remained on the study without receiving a non-study COVID-19 vaccination. One participant with a partial death date in the AZD1222 group was excluded from the all-cause mortality analysis. COVID-19, coronavirus disease 2019; FAS, full analysis set.

All-cause mortality and COVID-19-related mortality remained lower in the AZD1222 group compared with the placebo group at all timepoints, as summarized in Figure 2A and Figure 2B, respectively. However, the shorter follow-up duration in the placebo group coupled with the very low overall number of deaths recorded in the study precluded further analysis or interpretation of efficacy.

### 3.4. Long-Term Immunogenicity

**Figure 3:**
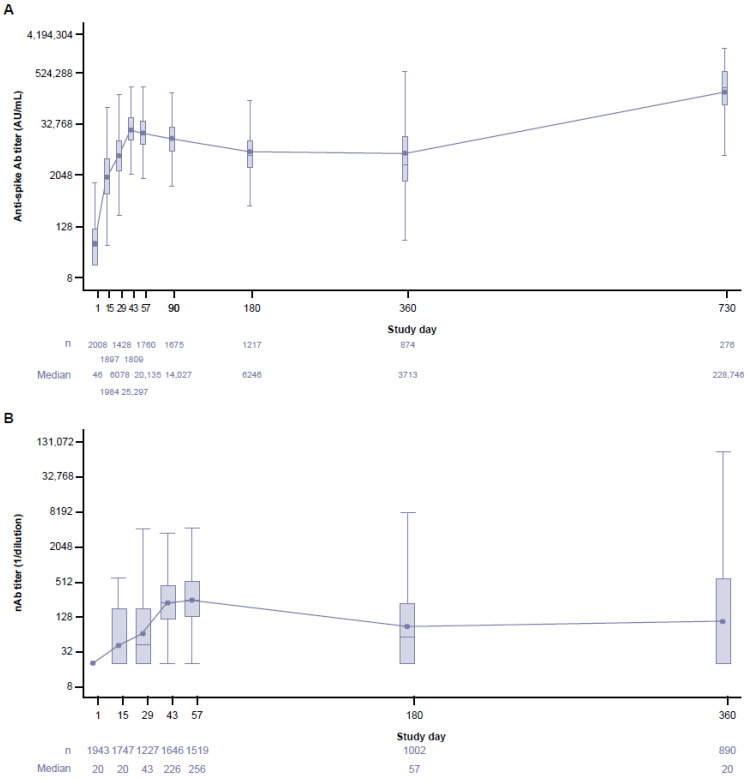
(**A**) Anti-spike Ab titers against ancestral SARS-CoV-2 in participants up to day 730 and (**B**) nAb titers against ancestral SARS-CoV-2 in participants up to day 360 in the AZD1222 group. Box and whisker plots show anti-spike Ab and nAb titers against ancestral SARS-CoV-2 over time in the immunogenicity substudy population; for timepoints after dosing (i.e., post-study day 29), only participants who had received two doses of AZD1222 and remained in the study for at least 15 days after dosing were included. The box denotes the IQR, the line inside the box denotes median, and the marker inside the box is the geometric mean. Any points >1.5 × IQR from the box were considered outliers and are not displayed. The whiskers that extend from the box indicate the minimum and maximum after removing the outliers. The boxplots are presented in a log2 scale. The baseline was defined as the last non-missing measurement taken prior to the first dose of AZD1222 (including unscheduled measurements, if any). Titers below the LLoQ were imputed to half of the LLoQ. Titers above the ULoQ were imputed to the ULoQ. Assessments collected after non-study COVID-19 vaccine administration/exclusionary restricted medication intake were excluded. Immunogenicity data up to study day 180 have been previously reported [11,16]. Ab, antibody; AU, arbitrary units; IQR, interquartile range; LLoQ, lower limit of quantification; nAb, neutralizing antibody; S, spike; ULoQ, upper limit of quantification.

Overall, AZD1222 was observed to generate a robust humoral immune response. In the AZD1222 group, an increase in anti-spike Ab titers against ancestral SARS-CoV-2 was detectable at day 15 (GMT = 1820.10 arbitrary units (AU)/mL [95% CI: 1696.48–1952.72]) then reached a peak at day 43 (GMT = 24,105.87 AU/mL [95% CI: 22,945.04–25,325.44]), 14 days after the second dose (Figure 3A). Anti-spike Ab levels were maintained through at least day 360 (GMT = 6686.81 AU/mL [95% CI: 5779.74–7736.24]). As expected, anti-spike responses waned from the titers induced at day 43 but remained above the titers observed after the first dose. An increase in anti-spike Ab levels was observed at day 730 in the AZD1222 group (GMT = 186,727.78 AU/mL [95% CI: 154,395.93–225,830.20]). In the placebo group, GMTs were also observed to increase at days 180, 360, and 730 (Figure S1A).

In the AZD1222 group, nAb responses against ancestral SARS-CoV-2 showed similar early kinetics to anti-spike Abs, with nAb titers peaking at day 57 (GMT = 250.6 [95% CI: 234.9–267.4]), 28 days after the second dose (Figure 3B). Although nAb titers waned from the initial peak, they remained elevated up to day 180 (GMT = 89.5 [95% CI: 80.0–100.1]) and were greater than titers observed after the first dose. An increase in nAb levels was observed at day 360 in the AZD1222 group (GMT = 108.2 [95% CI: 92.7–126.3]). In the placebo group, GMTs also increased at day 180 and day 360 (Figure S1B). 

## 4. Discussion

These data from the 2-year follow-up of the AZD1222 phase 3 trial confirm that the safety profile of AZD1222 primary series vaccination remains consistent with previous reports, and no new safety signals were observed. There were also no new safety signals in participants who received a non-study COVID-19 vaccination (i.e., a booster dose) post-AZD1222 [11,16]. Considering that more than 60% of people are vaccinated with a complete primary series worldwide, and AZD1222 is among the most widely used of the COVID-19 vaccines, these data on the extended safety of AZD1222 primary series vaccination are highly relevant to global vaccination strategies [6,29]. Additionally, the extended safety of AZD1222 is important for the ongoing development of the ChAdOx1 platform as a rapid response vaccine technology for future pandemic threats [15]. The fact that we observed no emergent safety signals in participants who received a booster dose after AZD1222 primary series offers further support for the safety of widely adopted heterologous booster strategies [8,30,31,32].

Reported in post-marketing data, TTS is an extremely rare but serious safety signal observed after COVID-19 vaccination [27,28]. We did not observe any cases of TTS during this large-scale clinical study. This is consistent with our findings throughout the clinical development of AZD1222, where TTS was also not observed [11,33]. As the estimated reporting rates of TTS following AZD1222 vaccination are 7.5 to 20 per million vaccinated persons and as exceptionally few incidences have been reported following a second-dose or third-dose booster [28,34,35], ongoing pharmacovigilance studies involving millions of individuals remain the most appropriate avenue for evaluating TTS etiology and dynamics. 

The high level of protection afforded by two doses of AZD1222 against SARS-CoV-2, with continued durability through 6 months, has been reported previously in this study [16]. Over the course of this 2-year follow-up, the incidence of seroconversion for anti-SARS-CoV-2 nucleocapsid Abs in AZD1222 group participants (i.e., detected SARS-CoV-2 infection) increased over time. In fact, it is likely that the incidence of SARS-CoV-2 infection was under-represented at the later timepoints due to the length of time between study visits (study day 360 to 730), which potentially allowed enough time for any interim elevation in anti-SARS-CoV-2 nucleocapsid Ab levels following natural infection to return to below threshold levels before the next study visit. The most notable increase in infections occurred after the 6-month interim analysis and coincided with the emergence and peak spread of the Delta variant in mid-2021, followed swiftly by Omicron BA.1 predominance in late 2021 [20]. Subsequent waves of Omicron subvariants, including robust circulation of BA.2, BA.2.12.1, BA.4, BA.5, BQ., XXB, and XXB.1.5, continued to dominate COVID-19 cases until the end of the study [20]. Recent real-world studies have observed a similar decrease in the protection afforded by vaccination against breakthrough infections and symptomatic disease during both Delta and Omicron predominance [12,36], a likely reflection of the increased transmissibility and immune evasion features inherent to the most recent SARS-CoV-2 variants [23].

Despite a notable increase in SARS-CoV-2 infections, mortality rates remained low throughout this study. The low number of overall deaths could be explained, in part, by the decoupling of infection rates and severe COVID-19 outcomes that has coincided with Omicron circulation, a trend hypothesized to have occurred for two possible reasons: (1) Omicron is associated with milder disease in the general population, although vulnerable individuals remain at increased risk; or (2) robust SARS-CoV-2 immunity has been established in global populations due to hybrid immunity (a combination of COVID-19 vaccination and natural infection) [37,38,39,40,41]. The protective effect of AZD1222 against severe COVID-19 outcomes has been demonstrated previously and likely contributed to the limited number of deaths from SARS-CoV-2 in the study population [13,16,42]. Indeed, all-cause and COVID-19-related mortality were numerically lower in the AZD1222 group than the placebo group at all time points. However, interpretations of these results are precluded by the shorter observed follow-up times in the placebo group. 

For analyses of extended immunogenicity, the full substudy population was assessed regardless of baseline serostatus for SARS-CoV-2 infection, with the intent to reflect the now widespread hybrid immunity known to exist in the general population [43]. The strong initial humoral response elicited by the AZD1222 primary series has been reported and interpreted previously [11,16]; however, in keeping with previous studies, our long-term immunogenicity follow-up demonstrated a waning in humoral immunogenicity [10,44]. Ab titers decreased from the peak up to day 360 for anti-spike Abs, and up to day 180 for nAbs, although responses remained above the level observed after the first dose. It is likely that this waning in humoral responses contributed to the increased incidence in SARS-CoV-2 infections observed after 6 months. A similar waning in protection against infection has been demonstrated following primary-series vaccination with other COVID-19 vaccines, including mRNA vaccines [45,46]. In addition to an increased risk of infection, it has previously been suggested that suboptimal nAb titer levels could increase the risk of Ab-dependent disease enhancement associated with COVID-19 vaccines [47]; however, we observed no evidence of vaccine-associated enhanced disease despite the observed waning in nAb responses.

Unexpectedly, we observed an apparent increase in humoral responses in both the AZD1222 group and the placebo group at day 730 for anti-spike Abs and at day 360 for nAbs. This could be explained by a potential under-reporting of non-study COVID-19 vaccines received, as discussed at the 6-month interim analysis [16]; however, an increase in SARS-CoV-2 infections, particularly undetected asymptomatic or mild infection driven by Omicron, likely contributed to the observed increase [37,38,39,40,41]. Indeed, this finding is consistent with other clinical studies from the same time period, in particular the AZD2816 (a Beta [B.1.351] variant COVID-19 vaccine) and AZD1222 clinical trials, which noted differences in immunogenicity over time, potentially due to Omicron circulation [30]. 

Taken together, our durability of protection and immunogenicity findings are consistent with clinical findings and real-world evidence, suggesting that high-level protection against symptomatic COVID-19 after AZD1222 primary-series vaccination persists for approximately 4–6 months. Furthermore, our results highlight the combined effect of waning immunity and the emergence of novel variants with increased transmissibility and immune evasion in COVID-19 cases [16,42]. These data confirm previously established findings that the AZD1222 primary series is effective at preventing infection for prior SARS-CoV-2 variants but is less effective against Omicron sub-lineage infections [12,13]. Other primary-series COVID-19 vaccinations have also been reported to have reduced efficacy against Omicron subvariants [12,13,14]. Recently, several real-world studies have demonstrated that repeated booster dosing with either COVID-19 vaccines developed against ancestral SARS-CoV-2 or variant-updated vaccines are effective at mitigating severe COVID-19 outcomes and mortality from Omicron [48,49,50,51,52,53,54]. Given that the elderly, immunocompromised, and those with underlying comorbidities remain at risk from severe outcomes from Omicron variant infection, our findings support the need for ongoing booster vaccinations to protect individuals vulnerable to severe disease, as recommended by the WHO [8,40,41]. 

Throughout the course of the study, there was extensive unblinding of participants once COVID-19 vaccines became available through EUAs in the USA, Chile, and Peru [4]. As would be expected, and consistent with observations at the primary and 6-month analyses, participants in the placebo group demonstrated greater non-study COVID-19 vaccine-seeking behavior [11,16]. The primary limitation of this analysis is the resultant short follow-up times and greater reduction of the at-risk population in the placebo group, which prohibited comparison between study groups for long-term endpoints. Additionally, COVID-19 boosters became widely available during follow-up, further restricting the population available for analyses of the primary-series AZD1222 [25,26]. This limitation epitomizes the challenge of running randomized, placebo-controlled studies while managing participant welfare in a pandemic setting. Other phase 3 studies were also limited by the ethical and practical need to immunize eligible placebo participants when EUAs were granted [55,56,57]. We are not aware of other 2-year analyses for widely used primary series COVID-19 vaccines, but they would also likely face similar challenges. 

Another limitation is that the current global population may have been more accurately represented by SARS-CoV-2 seropositive participants, as hybrid immunity is now widespread [43]; however, the low proportion of participants who were seropositive at baseline (2.8%) precluded an assessment of AZD1222 immunogenicity and duration of protection in the seropositive population. Finally, while demographics remained balanced between the AZD1222 and placebo groups, we cannot be sure that the population censored at non-study COVID-19 vaccination is reflective of the baseline study population, as they represent a self-selecting population that did not seek out non-study COVID-19 vaccinations.

## 5. Conclusions

This 2-year follow-up analysis provides the first large-scale, extended clinical study data for one of the most widely used COVID-19 vaccines, AZD1222 [6]. These findings reinforce that the long-term safety profile of the AZD1222 primary series remains consistent with previous reports, with no emergent safety issues detected [11,16]. AZD1222 elicits persistent humoral responses with evidence of waning after 6 months, thus supporting the continued use of COVID-19 booster vaccinations.

## Figures and Tables

**Table 1:** Summary of AEs prior to and after non-study COVID-19 vaccination in the AZD1222 group.

AEs, Participants (%) Events/Adj. Rate	AZD1222 (Safety Population)		
	Prior to Non-Study COVID-19 Vaccination N = 21,587 Patient Years = 20,223	After Non-Study COVID-19 Vaccination N = 14,667 Patient Years = 17,088	Overall N = 21,587 Patient Years = 37,311
AEs with outcome of death Related AEs with outcome of death	42 (0.2) 46/< 0.01 0	20 (0.1) 22/< 0.01 0	62 (0.3) 68/< 0.01 0
AEs leading to study discontinuation ^1^ Related AEs leading to study discontinuation ^1^	43 (0.2) 46/< 0.01 0	20 (0.1) 20/< 0.01 0	63 (0.3) 66/< 0.01 0
SAEs ^2^ Related SAEs ^2^	621 (2.9) 870/0.03 7 (<0.1) 9/< 0.01	456 (3.1) 622/0.03 0	1039 (4.8) 1492/0.03 7 (<0.1) 9/< 0.01
MAAEs ^2^ Related MAAEs ^2^	4750 (22.0) 8300/0.23 107 (0.5) 176/< 0.01	3344 (22.8) 5660/0.20 2 (<0.1) 2/< 0.01	6955 (32.2) 13,960/0.19 108 (0.5) 178/< 0.01
AESIs ^2^ Related AESIs ^2^	2516 (11.7) 2787/0.12 68 (0.3) 83/< 0.01	4369 (29.8) 4710/0.26 1 (<0.1) 1/< 0.01	6622 (30.7) 7497/0.18 68 (0.3) 84/< 0.1

AEs were assessed in the safety population, which included participants who received at least one dose of AZD1222 or placebo. The adj. rate was calculated as: (number of participants with AEs)/(total patient-year of observation). Patient years were calculated as: (total number of follow-up days for each participant in the AZD1222 group)/365.25. The exposure period ‘prior to non-study COVID-19 vaccination’ was calculated from the time of first dose of AZD1222 to the time of first non-study COVID-19 vaccination or the end of the study, whichever occurred first. The period ‘after non-study COVID-19 vaccination’ was calculated from the time of first non-study COVID-19 vaccination to the end of the study. Percentages were based on the number of participants in the safety analysis set in the AZD1222 group for each period. Participants missing non-study COVID-19 vaccination date or missing start date of event were classified as having the event prior to non-study COVID-19 vaccination. Participants with events that overlapped their non-study COVID-19 vaccination date were counted in both the prior and post-non-study COVID-19 vaccination subgroups. Related events were those that were considered related according to the investigator. These data provide an update to the safety data previously reported [3,4]. ^1^ SAEs, MAAEs, and AESIs leading to discontinuation were reported for the duration of the study; non-serious AEs leading to discontinuation were captured only up to day 57. ^2^ SAEs, MAAEs, and AESIs were recorded from the time of informed consent through to the last participant contact. Adj. rate, exposure-adjusted rate; AE, adverse event; AESI, adverse event of special interest; COVID-19, coronavirus disease 2019; MAAE, medically attended adverse event; SAE, serious adverse event.

**Table 2:** Incidence of first positive response for anti-SARS-CoV-2 nucleocapsid Abs occurring from day 15 after the second dose in the AZD1222 group.

Time Period	AZD1222 (FVAS, Censored at Non-Study COVID-19 Vaccination)		
	n/N (%) ^1^	Follow-Up Time ^2^	Incidence Rate ^2^
≥15 days post-second dose	2925/19,409 (15.1)	15.47	189.05
≥15 days post-second dose to <6 months post-first dose	421/19,409 (2.2)	6.31	66.72
≥6 months post-first dose	2504/14,520 (17.2)	9.16	273.28
≥1 year post-first dose	1678/6850 (24.5)	3.44	487.90

Response for anti-SARS-CoV-2 nucleocapsid Abs was assessed in the FVAS population, censored at non-study COVID-19 vaccination. The FVAS included all participants who were SARS-CoV-2 seronegative at baseline, received both doses, and remained in the study for ≥15 days post-second dose without prior confirmed SARS-CoV-2 RT-PCR-positive infection. Participants who received a non-study COVID-19 vaccination prior to 15 days post-second dose were excluded from the analysis set. Participants who received a non-study COVID-19 vaccination ≥15 days post-second dose were censored at the date of non-study COVID-19 vaccination. ^1^ One participant excluded from the FVAS in error has not been corrected for in this table. ^2^ Follow-up time and incidence rate are presented per 1000 person-years. Ab, antibody; COVID-19, coronavirus disease 2019; FVAS, fully vaccinated analysis set; RT-PCR, reverse transcription polymerase chain reaction; SARS-CoV-2, severe acute respiratory syndrome coronavirus 2.

## Data Availability

Data underlying the findings described in this manuscript may be obtained in accordance with AstraZeneca’s data sharing policy described at https://astrazenecagrouptrials.pharmacm.com/ST/Submission/Disclosure (accessed on 23 October 2023). Data for studies directly listed on Vivli can be requested through Vivli at www.vivli.org (accessed on 23 October 2023). Data for studies not listed on Vivli can be requested through Vivli at https://vivli.org/members/enquiries-about-studies-not-listed-on-the-vivli-platform/ (accessed on 23 October 2023). The AstraZeneca Vivli member page is also available, outlining further details: https://vivli.org/ourmember/astrazeneca/ (accessed on 23 October 2023).

## Supplementary Materials

The following supporting information can be downloaded at: https://www.mdpi.com/article/10.3390/vaccines12080883/s1, Supplementary Methods; Figure S1: (A) Anti-SARS-CoV-2 spike Ab in participants up to day 730 and (B) anti-SARS-CoV-2 nAb titers in participants up to day 360, in the placebo group; Table S1: Summary of follow-up times and at-risk participants in the safety population after first and second doses of AZD1222 or placebo over the study duration; Table S2: Participant demographics and clinical characteristics in key analysis populations; Table S3: Summary of adverse events prior to and after non-study COVID-19 vaccination in the placebo group for the duration of the study; Table S4: Incidence of first positive response for anti-SARS-CoV-2 nucleocapsid Abs occurring from day 15 after the second dose in the placebo group; Table S5: Independent ethics committee or institutional review board approvals.
