# Supplementary material for: Long-Term Safety and Immunogenicity of AZD1222 (ChAdOx1 nCoV-19): 2-Year Follow-Up from a Phase 3 Study
Source: Vaccines (Basel). 2024 Aug 3;12(8):883. doi: 10.3390/vaccines12080883 (PMC11359581; doi:10.3390/vaccines12080883)
Supplement: Supplementary file 1 [file vaccines-12-00883-s001.zip › vaccines-3086345-supplementary.pdf]

vaccines

MDPI

Supplementary Material

Long-Term Safety and Immunogenicity of AZD1222 (ChAdOx1 nCoV-19): 2-Year Follow-Up from a Phase 3 study

Kathryn Shoemaker, Karina Soboleva, Angela Branche, Shivanjali Shankaran, Deborah A. Theodore, Muhammad Bari, Victor Ezeh, Justin Green, Elizabeth Kelly, Dongmei Lan, Urban Olsson, Senthilkumar Saminathan, Nirmal Kumar Shankar, Berta Villegas, Tonya Villafana, Ann R. Falsey and Magdalena E. Sobieszczyk

List of supplementary materials

	Page
**Supplementary Methods**	2
**Supplementary Figures**	3
**Figure S1**. (**A**) Anti-SARS-CoV-2 spike Ab titers in participants up to day 730 and (**B**) anti-SARS-CoV-2 nAb titers in participants up to day 360, in the placebo group	3
**Supplementary Tables**	4
**Table S1.** Summary of follow-up times and at-risk participants in the safety population after first and second doses of AZD1222 or placebo over the study duration	4
**Table S2.** Participant demographics and clinical characteristics in key analysis populations	6
**Table S3.** Summary of adverse events prior to and after non-study COVID-19 vaccination in the placebo group for the duration of the study	10
**Table S4.** Incidence of first positive response for anti-SARS-CoV-2 nucleocapsid Abs occurring from day 15 after the second dose in the placebo group	11
**Table S5.** Independent ethics committee or institutional review board approvals	12
**Supplementary References**	18

Supplementary Methods

Serological Assessments

Response for anti-SARS-CoV-2 nucleocapsid antibodies (Abs) was qualitatively measured for all participants with a validated Roche Elecsys® anti-severe acute respiratory syndrome coronavirus 2 (SARS-CoV-2) nucleocapsid serology test (Covance CLS, Indianapolis, IN, USA).

Multiplex serology immunoglobulin G (IgG) assays [1] were used to quantitatively assess serum levels of anti-spike Abs against ancestral SARS-CoV-2, as described previously [2-4]. All analyses were performed at PPD Vaccines (Richmond, VA, USA) in validated assays. Briefly, a Meso Scale Discovery® platform (Meso Scale Diagnostics LLC, Rockville, MD, USA) was used to determine Ab concentrations through indirect binding. Relative light unit outputs were then interpolated relative to a standard curve generated from a serially diluted reference standard (pooled COVID-19-positive serum samples) and assigned a concentration of arbitrary units (AU)/mL.

Pseudovirus neutralization assays were used to determine neutralizing antibody (nAb) titers against ancestral SARS-CoV-2 in serum, as described previously [2-4]. All analyses were performed at Monogram Biosciences (South San Francisco, CA, USA) in validated assays. Briefly, pseudovirions containing luciferase and an ancestral SARS-CoV-2 virus spike protein were preincubated with serial dilutions of serum. SARS-CoV-2 nAb titers were reported as the reciprocal of the serum dilution equivalent to the ID_{50} of pseudovirus infection (a fifty percent reduction of luciferase reporter signal).

Supplementary Figures

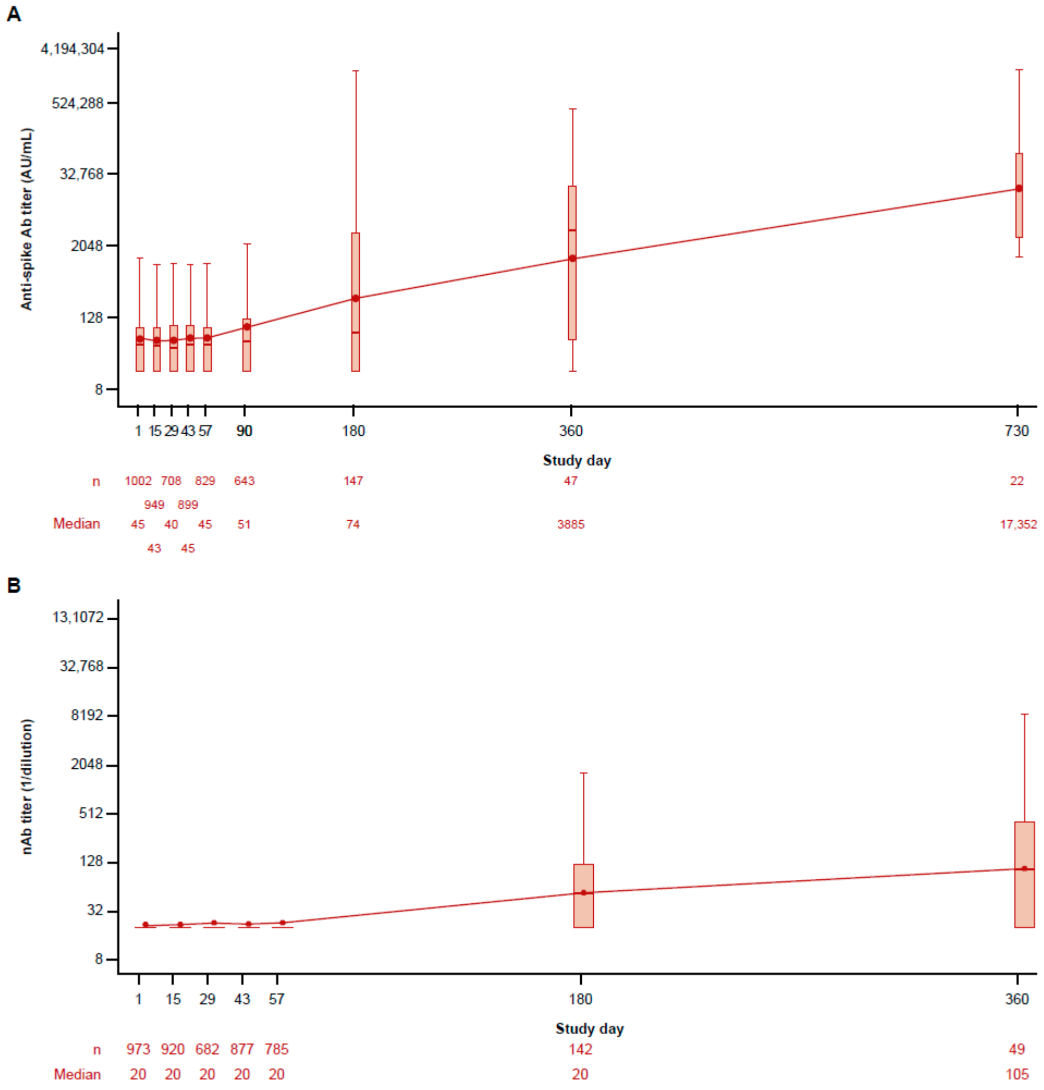

Figure S1. (**A**) Anti-spike Ab titers against ancestral SARS-CoV-2 in participants up to day 730 and (**B**) nAb titers against ancestral SARS-CoV-2 in participants up to day 360, in the placebo group. Box and Whisker plots show anti-spike Ab and nAb titers against ancestral SARS-CoV-2 over time in the immunogenicity substudy population; for timepoints after dosing (i.e., post-study day 29), only participants who had received two doses of placebo and remained in the study for at least 15 days after dosing were included. The box denotes the IQR, the line inside the box denotes median and the marker inside the box is the geometric mean. Any points >1.5×IQR from the box were considered outliers and are not displayed. The whiskers that extend from the box indicate the minimum and maximum after removing the outliers. The boxplots are presented in log2 scale. Baseline was defined as the last non-missing measurement taken prior to the first dose of placebo (including unscheduled measurements, if any). Titers below the LLoQ were imputed to half of the LLoQ. Titers above the ULoQ were imputed to the ULoQ. Assessments collected after non-study COVID-19 vaccine administration/exclusionary restricted medication intake were excluded. Immunogenicity data up to study day 180 have been previously reported [3,4]. Ab, antibody; AU, arbitrary units; COVID-19, coronavirus disease 2019; IQR, interquartile range; LLoQ, lower limit of quantification; nAb, neutralizing antibody; SARS-CoV-2, severe acute respiratory syndrome coronavirus 2; ULoQ, upper limit of quantification.

Supplementary Tables

Participants at risk	AZD1222<br>N = 21,587	Placebo<br>N = 10,793	Total<br>N = 32,380
**Safety population, whole study period**			
Duration of follow up, days (min, max)			
Median follow-up after first dose	714.0 (1, 864)	710.0 (1, 839)	713.0 (1, 864)
Median follow-up after second dose	686.0 (1, 820)	682.0 (1, 780)	685.0 (1, 820)
Participants at risk from first dose, n (%)			
Month 1 (D30)	21,587 (100.0)	10,793 (100.0)	32,380 (100.0)
Month 6 (D180)	20,323 (94.1)	9715 (90.0)	30,038 (92.8)
Month 8 (D240)	19,660 (91.1)	9104 (84.4)	28,764 (88.8)
Month 12 (D360)	19,299 (89.4)	8835 (81.9)	28,134 (86.9)
Month 24 (D720)	17,204 (79.7)	7738 (71.7)	24,942 (77.0)
Participants at risk from second dose, n (%)			
Month 1 (D30)	20,774 (96.2)	9950 (92.2)	30,724 (94.9)
Month 6 (D180)	19,605 (90.8)	8902 (82.5)	28,507 (88.0)
Month 8 (D240)	19,079 (88.4)	8465 (78.4)	27544 (85.1)
Month 12 (D360)	18,336 (84.9)	8070 (74.8)	26,406 (81.6)
Month 24 (D720)	9129 (42.3)	4048 (37.5)	13,177 (40.7)
**Safety population, period up to non-study COVID-19 vaccination**			
Duration of follow up, days (min, max)			
Median follow-up after first dose	328.0 (1, 848)	100.0 (1, 789)	207.0 (1, 848)
Median follow-up after second dose	304.0 (1, 820)	75.0 (1, 754)	200.0 (1, 820)
Participants at risk from first dose, n (%)			
Month 1 (D30)	21,587 (100.0)	10,793 (100.0)	32,380 (100.0)
Month 6 (D180)	16,635 (77.1)	2474 (22.9)	19,109 (59.0)

Month 8 (D240)	14,889 (69.0)	1172 (10.9)	16,061 (49.6)
Month 12 (D360)	10,634 (49.3)	606 (5.6)	11,240 (34.7)
Month 24 (D720)	3718 (17.2)	335 (3.1)	4053 (12.5)
Participants at risk from second dose, n (%)			
Month 1 (D30)	20,750 (96.1)	9908 (91.8)	30,658 (94.7)
Month 6 (D180)	15,696 (72.7)	1638 (15.2)	17,334 (53.5)
Month 8 (D240)	14,180 (65.7)	898 (8.3)	15,078 (46.6)
Month 12 (D360)	8321 (38.5)	511 (4.7)	8832 (27.3)
Month 24 (D720)	2029 (9.4)	178 (1.6)	2207 (6.8)

Table S1. Summary of follow-up times and at-risk participants in the safety population after first and second doses of AZD1222 or placebo over the study duration. For the 'whole study period', follow-up times were censored at study discontinuation or end of study, regardless of non-study COVID-19 vaccination. For the 'period to non-study COVID-19 vaccination', participants were also censored at non-study COVID-19 vaccination. For participants with non-study COVID-19 vaccination date before first dose of study intervention, the follow-up time was imputed to 1. The safety population included participants who received at least one dose of AZD1222 or placebo. Percentages were based on the number of participants in the safety population by study group. One participant with partial death date in the AZD1222 group was censored at the data cut-off (March 21, 2023). These data provide an update to the follow-up times previously reported [3,4]. COVID-19, coronavirus disease 2019, DX, day X.

Characteristic	AZD1222	Placebo	Total
**Safety population per actual study intervention**	**N = 21,587**	**N = 10,793**	**N = 32,380**
Age, median (range)	51.0 (18–100)	51.0 (18–92)	51.0 (18–100)
≥18 to <65 years, n (%)	16,759 (77.6)	8382 (77.7)	25,141 (77.6)
≥65 years, n (%)	4828 (22.4)	2411 (22.3)	7239 (22.4)
Sex, n (%)			
Male	12,009 (55.6)	6004 (55.6)	18,013 (55.6)
Female	9578 (44.4)	4789 (44.4)	14,367 (44.4)
Race, n (%)			
White	17,062 (79.0)	8523 (79.0)	25,585 (79.0)
Black or African American	1793 (8.3)	892 (8.3)	2685 (8.3)
Asian	947 (4.4)	482 (4.5)	1429 (4.4)
American Indian/Alaska Native	853 (4.0)	428 (4.0)	1281 (4.0)
Native Hawaiian or Other Pacific Islander	60 (0.3)	21 (0.2)	81 (0.3)
Multiple[1]	511 (2.4)	257 (2.4)	768 (2.4)
Unknown or not reported	361 (1.7)	190 (1.8)	551 (1.7)
Hispanic/Latino, n (%)			
Yes	4771 (22.1)	2451 (22.7)	7222 (22.3)
No	16,475 (76.3)	8202 (76.0)	24,677 (76.2)
Unknown or not reported	341 (1.6)	140 (1.3)	481 (1.5)
Enrolment country, n (%)			
USA	19,145 (88.7)	9573 (88.7)	28,718 (88.7)
Chile	1470 (6.8)	729 (6.8)	2199 (6.8)
Peru	972 (4.5)	491 (4.5)	1463 (4.5)
Baseline SARS-CoV-2 serostatus[2], n (%)			
Negative	20,694 (95.9)	10,349 (95.9)	31,043 (95.9)
Positive	623 (2.9)	293 (2.7)	916 (2.8)
Missing or not done	269 (1.2)	149 (1.4)	418 (1.3)
Baseline COVID-19 comorbidities[3], n (%)			
Yes	12,939 (59.9)	6498 (60.2)	19,437 (60.0)
No	8646 (40.1)	4294 (39.8)	12,940 (40.0)

**FVAS, censored at non-study COVID-19 vaccination**	**N = 19,529**	**N = 8838**	**N = 28,367**
Age, median (range)	51.0 (18–99)	51.0 (18–91)	51.0 (18–99)
≥18 to <65 years, n (%)	15,075 (77.2)	6892 (78.0)	21,967 (77.4)
≥65 years, n (%)	4454 (22.8)	1946 (22.0)	6400 (22.6)
Sex, n (%)			
Male	10,806 (55.3)	4958 (56.1)	15,764 (55.6)
Female	8723 (44.7)	3880 (43.9)	12,603 (44.4)
Race, n (%)			
White	15,559 (79.7)	6998 (79.2)	22,557 (79.5)
Black or African American	1529 (7.8)	715 (8.1)	2244 (7.9)
Asian	850 (4.4)	368 (4.2)	1218 (4.3)
American Indian/Alaska Native	764 (3.9)	375 (4.2)	1139 (4.0)
Native Hawaiian or Other Pacific Islander	54 (0.3)	18 (0.2)	72 (0.3)
Multiple[1]	452 (2.3)	210 (2.4)	662 (2.3)
Unknown or not reported	321 (1.6)	154 (1.7)	475 (1.7)
Hispanic/Latino, n (%)			
Yes	4310 (22.1)	2100 (23.8)	6410 (22.6)
No	14,922 (76.4)	6620 (74.9)	21,542 (75.9)
Unknown or not reported	297 (1.5)	118 (1.3)	415 (1.5)
Enrolment country, n (%)			
USA	17,245 (88.3)	7722 (87.4)	24,967 (88.0)
Chile	1410 (7.2)	681 (7.7)	2091 (7.4)
Peru	874 (4.5)	435 (4.9)	1309 (4.6)
Baseline SARS-CoV-2 serostatus[2], n (%)			
Negative	19,529 (100)	8838 (100)	28,367 (100)
Positive	0	0	0
Missing or not done	0	0	0
Baseline COVID-19 comorbidities[3], n (%)			
Yes	11,688 (59.9)	5342 (60.5)	17,030 (60.0)
No	7839 (40.1)	3495 (39.5)	11,334 (40.0)

**Immunogenicity substudy per actual study intervention, censored at non-study COVID-19 vaccination**	**N = 2025**	**N = 1009**	**N = 3034**
Age, median (range)	56.0 (18–101)	55.0 (18–90)	56.0 (18–101)
≥18 to <65 years, n (%)	1330 (65.7)	660 (65.4)	1990 (65.6)
≥65 years, n (%)	695 (34.3)	349 (34.6)	1044 (34.4)
Sex, n (%)			
Male	1211 (59.8)	580 (57.5)	1791 (59.0)
Female	814 (40.2)	429 (42.5)	1243 (41.0)
Race, n (%)			
White	1802 (89.0)	900 (89.2)	2702 (89.1)
Black or African American	105 (5.2)	59 (5.8)	164 (5.4)
Asian	53 (2.6)	19 (1.9)	72 (2.4)
American Indian/Alaska Native	13 (0.6)	10 (1.0)	23 (0.8)
Native Hawaiian or Other Pacific Islander	3 (0.1)	1 (0.1)	4 (0.1)
Multiple[1]	26 (1.3)	11 (1.1)	37 (1.2)
Unknown or not reported	23 (1.1)	9 (0.9)	32 (1.1)
Hispanic/Latino, n (%)			
Yes	170 (8.4)	87 (8.6)	257 (8.5)
No	1807 (89.2)	909 (90.1)	2716 (89.5)
Unknown or not reported	48 (2.4)	13 (1.3)	61 (2.0)
Enrolment country, n (%)			
USA	2025 (100.0)	1009 (100.0)	3034 (100.0)
Chile	0	0	0
Peru	0	0	0
Baseline SARS-CoV-2 serostatus[2], n (%)			
Negative	1983 (97.9)	973 (96.4)	2956 (97.4)
Positive	20 (1.0)	22 (2.2)	42 (1.4)
Missing or not done	22 (1.1)	14 (1.4)	36 (1.2)
Baseline COVID-19 comorbidities[3], n (%)			
Yes	1279 (63.2)	653 (64.7)	1932 (63.7)
No	746 (36.8)	356 (35.3)	1102 (36.3)

Table S2. Participant demographics and clinical characteristics in key analysis population. The safety population included participants who received at least one dose of AZD1222 or placebo. The FVAS included all participants who were SARS-CoV-2 seronegative at baseline, received both doses, and remained in the study for ≥15 days post second dose without prior confirmed SARS-CoV-2 RT-PCR-positive infection. These data provide an update to the participant characteristics previously reported [3,4]. [1]Participants who reported more than one race are reported under 'Multiple'. [2]Serostatus at baseline was defined by the nucleocapsid Ab level as measured by the Elecsys Anti-SARS-CoV-2 serology test (Roche). [3]Defined as participants with medical conditions that may place them at a higher risk for acquisition of, or more severe, COVID-19 disease, as described previously [3]. Ab, antibody; COVID-19, coronavirus disease 2019; FVAS, fully vaccinated analysis set; RT-PCR, reverse transcription-polymerase chain reaction; SARS-CoV-2, severe acute respiratory syndrome coronavirus 2.

AE, Participants (%) Events / Adj. Rate	Placebo (safety population)		
	Prior to non-study COVID-19 vaccination N = 10,793 Patient years = 3893	After non-study COVID-19 vaccination N = 9074 Patient years = 13,404	Overall N = 10,793 Patient years = 17,297
AEs with outcome of death	14 (0.1) 17 / <0.01	19 (0.2) 21 / <0.01	33 (0.3) 38 / <0.01
Related AEs with outcome of death	0	0	0
AEs leading to study discontinuation[1]	14 (0.1) 15 / <0.01	19 (0.2) 20 / <0.01	33 (0.3) 35 / <0.01
Related AEs leading to study discontinuation[1]	0	0	0
SAEs[2]	136 (1.3) 180 / 0.03	339 (3.7) 449 / 0.03	467 (4.3) 629 / 0.03
Related SAEs[2]	1 (<0.1) 1 / <0.01	0	1 (<0.1) 1 / <0.01
MAAEs[2]	1256 (11.6) 1828 / 0.32	2231 (24.6) 4049 / 0.17	3131 (29.0) 5877 / 0.18
Related MAAEs[2]	31 (0.3) 45 / <0.01	1 (<0.1) 1 / <0.01	32 (0.3) 46 / <0.01
AESIs[2]	591 (5.5) 649 / 0.15	2398 (26.4) 2603 / 0.18	2905 (26.9) 3252 / 0.17
Related AESIs[2]	27 (0.3) 35 / <0.01	0	27 (0.3) 35 / <0.01

Table S3. Summary of AEs prior to and after non-study COVID-19 vaccination in the placebo group. AEs were assessed in the safety population, which included participants who received at least one dose of AZD1222 or placebo. Adj. rate was calculated as: (number of participants with AEs) / (total patient-year of observation). Patient-years were calculated as: (total number of follow-up days for each participant in the placebo group) / 365.25. The exposure period 'prior to non-study COVID-19 vaccination' was calculated from the time of first dose of placebo to the time of first non-study COVID-19 vaccination or the end of the study, whichever occurred first. The period 'after non-study COVID-19 vaccination' was calculated from the time of first non-study COVID-19 vaccination to the end of the study. Percentages were based on the number of participants in the safety analysis set in the placebo group for each period. Participants missing non-study COVID-19 vaccination date or missing start date of event were classified as having the event prior to non-study COVID-19 vaccination. Participants with events that overlapped their non-study COVID-19 vaccination date were counted in both the prior to and post-non-study COVID-19 vaccination subgroups. Related events were those considered related according to the investigator. These data provide an update to the safety data previously reported [3,4]. [1]SAEs, MAAEs, and AESIs leading to discontinuation were reported for the duration of the study; non-serious AEs leading to discontinuation were only captured up to day 57. [2]SAEs, MAAEs, and AESIs were recorded from the time of informed consent through to the last participant contact. Adj. rate, exposure-adjusted rate; AE, adverse event; AESI, adverse event of special interest; COVID-19, coronavirus disease 2019; MAAE, medically attended adverse event; SAE, serious adverse event.

Time Period	Placebo (FVAS, censored at non-study COVID-19 vaccination)		
	n/N (%)[1]	Follow-up time[2]	Incidence rate[2]
≥15 days post second dose	559/8782 (6.4)	2.19	255.49
≥15 days post second dose to <6 months post-first dose	356/8782 (4.1)	1.64	217.15
≥6 months post first dose	203/1367 (14.9)	0.55	370.09
≥1 year post first dose	121/359 (33.7)	0.22	539.37

Table S4. Incidence of first positive response for anti-SARS-CoV-2 nucleocapsid Abs occurring from day 15 after the second dose in the placebo group. Response for anti-SARS-CoV-2 nucleocapsid Abs was assessed in the FVAS population, censored at non-study COVID-19 vaccination. The FVAS included all participants who were SARS-CoV-2 seronegative at baseline, received both doses, and remained in the study for ≥15 days post second dose without prior confirmed SARS-CoV-2 RT-PCR-positive infection. Participants who received a non-study COVID-19 vaccination prior to 15 days post-second dose were excluded from the analysis set. Participants who received a non-study COVID-19 vaccination ≥15 days post second dose were censored at the date of non-study COVID-19 vaccination. [1]One participant excluded from the FVAS in error has not been corrected for in this table. [2]Follow-up time and incidence rate are presented per 1000 person-years. Ab, antibody; COVID-19, coronavirus disease 2019; FVAS, fully vaccinated analysis set; RT-PCR, reverse transcription-polymerase chain reaction; SARS-CoV-2, severe acute respiratory syndrome coronavirus 2.

Country	Centre no.	Name and address of IEC/IRB	Date of Approval
Chile	2005366	Universidad de Chile – Facultad de Medicina Independencia 1027, Santiago, Chile	30 October 2020
Chile	2005367	Universidad de Chile – Facultad de Medicina Independencia 1027, Santiago, Chile	12 November 2020
Chile	2005368	Universidad de Chile – Facultad de Medicina Independencia 1027, Santiago, Chile	19 November 2020
Peru	2005370	El Comite Nacional Transitoria de Etica en Investigacion Para la Evaluacion y Supervision Etica de los Ensayos Clinicos de la Enfermedad Cápac Yupanqui 1400 – Jesús María, Lima 11, Lima, Perú	17 September 2020
Peru	2005397	El Comite Nacional Transitoria de Etica en Investigacion Para la Evaluacion y Supervision Etica de los Ensayos Clinicos de la Enfermedad Cápac Yupanqui 1400 – Jesús María, Lima 11, Lima, Perú	15 October 2020
Peru	2005398	El Comite Nacional Transitoria de Etica en Investigacion Para la Evaluacion y Supervision Etica de los Ensayos Clinicos de la Enfermedad Cápac Yupanqui 1400 – Jesús María, Lima 11, Lima, Perú	21 October 2020
USA	2005258	WCGIRB – 1019 39th Ave., SE Suite 120, Puyallup, WA 98374, United States	07 August 2020
USA	2005259	WCGIRB – 1019 39th Ave., SE Suite 120, Puyallup, WA 98374, United States	19 August 2020
USA	2005260	WCGIRB – 1019 39th Ave., SE Suite 120, Puyallup, WA 98374, United States	01 September 2020
USA	2005261	WCGIRB – 1019 39th Ave., SE Suite 120, Puyallup, WA 98374, United States	10 August 2020
USA	2005264	WCGIRB – 1019 39th Ave., SE Suite 120, Puyallup, WA 98374, United States	31 August 2020
USA	2005265	WCGIRB – 1019 39th Ave., SE Suite 120, Puyallup, WA 98374, United States	24 August 2020
USA	2005266	WCGIRB – 1019 39th Ave., SE Suite 120, Puyallup, WA 98374, United States	07 August 2020
USA	2005267	WCGIRB – 1019 39th Ave., SE Suite 120, Puyallup, WA 98374, United States	21 August 2020
USA	2005268	WCGIRB – 1019 39th Ave., SE Suite 120, Puyallup, WA 98374, United States	03 September 2020

Country	Centre no.	Name and address of IEC/IRB	Date of Approval
USA	2005269	WCGIRB – 1019 39th Ave., SE Suite 120, Puyallup, WA 98374, United States	19 August 2020
USA	2005270	WCGIRB – 1019 39th Ave., SE Suite 120, Puyallup, WA 98374, United States	28 August 2020
USA	2005271	WCGIRB – 1019 39th Ave., SE Suite 120, Puyallup, WA 98374, United States	23 September 2020
USA	2005272	Oregon Health & Science University – 3181 SW Sam Jackson Park Rd, Portland, OR 97239, United States	25 September 2020
USA	2005273	WCGIRB – 1019 39th Ave., SE Suite 120, Puyallup, WA 98374, United States	18 August 2020
USA	2005274	WCGIRB – 1019 39th Ave., SE Suite 120, Puyallup, WA 98374, United States	10 August 2020
USA	2005275	WCGIRB – 1019 39th Ave., SE Suite 120, Puyallup, WA 98374, United States	13 August 2020
USA	2005276	WCGIRB – 1019 39th Ave., SE Suite 120, Puyallup, WA 98374, United States	24 August 2020
USA	2005277	WCGIRB – 1019 39th Ave., SE Suite 120, Puyallup, WA 98374, United States	28 August 2020
USA	2005280	WCGIRB – 1019 39th Ave., SE Suite 120, Puyallup, WA 98374, United States	10 August 2020
USA	2005281	WCGIRB – 1019 39th Ave., SE Suite 120, Puyallup, WA 98374, United States	14 August 2020
USA	2005282	WCGIRB – 1019 39th Ave., SE Suite 120, Puyallup, WA 98374, United States	31 August 2020
USA	2005283	WCGIRB – 1019 39th Ave., SE Suite 120, Puyallup, WA 98374, United States	07 August 2020
USA	2005284	WCGIRB – 1019 39th Ave., SE Suite 120, Puyallup, WA 98374, United States	21 August 2020
USA	2005285	WCGIRB – 1019 39th Ave., SE Suite 120, Puyallup, WA 98374, United States	21 August 2020
USA	2005286	WCGIRB – 1019 39th Ave., SE Suite 120, Puyallup, WA 98374, United States	19 August 2020
USA	2005288	WCGIRB – 1019 39th Ave., SE Suite 120, Puyallup, WA 98374, United States	10 August 2020

Country	Centre no.	Name and address of IEC/IRB	Date of Approval
USA	2005289	Sutter Health Institutional Review Board – 2121 N. California Blvd., Suite 310, Walnut Creek, CA 94596, United States	28 August 2020
USA	2005290	WCGIRB – 1019 39th Ave., SE Suite 120, Puyallup, WA 98374, United States	24 August 2020
USA	2005291	WCGIRB – 1019 39th Ave., SE Suite 120, Puyallup, WA 98374, United States	10 August 2020
USA	2005292	WCGIRB – 1019 39th Ave., SE Suite 120, Puyallup, WA 98374, United States	12 August 2020
USA	2005293	WCGIRB – 1019 39th Ave., SE Suite 120, Puyallup, WA 98374, United States	18 September 2020
USA	2005294	WCGIRB – 1019 39th Ave., SE Suite 120, Puyallup, WA 98374, United States	14 September 2020
USA	2005295	WCGIRB – 1019 39th Ave., SE Suite 120, Puyallup, WA 98374, United States	02 September 2020
USA	2005296	WCGIRB – 1019 39th Ave., SE Suite 120, Puyallup, WA 98374, United States	19 August 2020
USA	2005297	WCGIRB – 1019 39th Ave., SE Suite 120, Puyallup, WA 98374, United States	08 September 2020
USA	2005298	Research Protections Office – 85 South Prospect St, 213 Waterman Bldg, Burlington, VT 05402, United States	25 September 2020
USA	2005299	The Ohio State Biomedical Sciences Institutional Review Board – 1960 Kenny Road, Columbus, OH 43210, United States	07 September 2020
USA	2005300	WCGIRB – 1019 39th Ave., SE Suite 120, Puyallup, WA 98374, United States	08 September 2020
USA	2005303	WCGIRB – 1019 39th Ave., SE Suite 120, Puyallup, WA 98374, United States	13 August 2020
USA	2005304	WCGIRB – 1019 39th Ave., SE Suite 120, Puyallup, WA 98374, United States	27 August 2020
USA	2005305	WCGIRB – 1019 39th Ave., SE Suite 120, Puyallup, WA 98374, United States	21 August 2020
USA	2005306	WCGIRB – 1019 39th Ave., SE Suite 120, Puyallup, WA 98374, United States	18 August 2020
USA	2005307	WCGIRB – 1019 39th Ave., SE Suite 120, Puyallup, WA 98374, United States	19 August 2020

Country	Centre no.	Name and address of IEC/IRB	Date of Approval
USA	2005308	WCGIRB – 1019 39th Ave., SE Suite 120, Puyallup, WA 98374, United States	12 August 2020
USA	2005309	WCGIRB – 1019 39th Ave., SE Suite 120, Puyallup, WA 98374, United States	02 September 2020
USA	2005310	WCGIRB – 1019 39th Ave., SE Suite 120, Puyallup, WA 98374, United States	18 August 2020
USA	2005312	WCGIRB – 1019 39th Ave., SE Suite 120, Puyallup, WA 98374, United States	17 August 2020
USA	2005313	WCGIRB – 1019 39th Ave., SE Suite 120, Puyallup, WA 98374, United States	25 August 2020
USA	2005314	WCGIRB – 1019 39th Ave., SE Suite 120, Puyallup, WA 98374, United States	13 August 2020
USA	2005315	WCGIRB – 1019 39th Ave., SE Suite 120, Puyallup, WA 98374, United States	07 August 2020
USA	2005316	WCGIRB – 1019 39th Ave., SE Suite 120, Puyallup, WA 98374, United States	19 August 2020
USA	2005318	WCGIRB – 1019 39th Ave., SE Suite 120, Puyallup, WA 98374, United States	07 August 2020
USA	2005320	WCGIRB – 1019 39th Ave., SE Suite 120, Puyallup, WA 98374, United States	07 August 2020
USA	2005321	WCGIRB – 1019 39th Ave., SE Suite 120, Puyallup, WA 98374, United States	21 August 2020
USA	2005322	WCGIRB – 1019 39th Ave., SE Suite 120, Puyallup, WA 98374, United States	14 August 2020
USA	2005323	WCGIRB – 1019 39th Ave., SE Suite 120, Puyallup, WA 98374, United States	19 August 2020
USA	2005324	WCGIRB – 1019 39th Ave., SE Suite 120, Puyallup, WA 98374, United States	11 August 2020
USA	2005326	WCGIRB – 1019 39th Ave., SE Suite 120, Puyallup, WA 98374, United States	24 August 2020
USA	2005327	WCGIRB – 1019 39th Ave., SE Suite 120, Puyallup, WA 98374, United States	10 August 2020
USA	2005328	WCGIRB – 1019 39th Ave., SE Suite 120, Puyallup, WA 98374, United States	21 August 2020
USA	2005329	WCGIRB – 1019 39th Ave., SE Suite 120, Puyallup, WA 98374, United States	21 August 2020

Country	Centre no.	Name and address of IEC/IRB	Date of Approval
USA	2005330	WCGIRB – 1019 39th Ave., SE Suite 120, Puyallup, WA 98374, United States	14 August 2020
USA	2005332	WCGIRB – 1019 39th Ave., SE Suite 120, Puyallup, WA 98374, United States	24 August 2020
USA	2005333	WCGIRB – 1019 39th Ave., SE Suite 120, Puyallup, WA 98374, United States	14 August 2020
USA	2005335	WCGIRB – 1019 39th Ave., SE Suite 120, Puyallup, WA 98374, United States	20 August 2020
USA	2005336	WCGIRB – 1019 39th Ave., SE Suite 120, Puyallup, WA 98374, United States	10 August 2020
USA	2005347	WCGIRB – 1019 39th Ave., SE Suite 120, Puyallup, WA 98374, United States	27 August 2020
USA	2005348	WCGIRB – 1019 39th Ave., SE Suite 120, Puyallup, WA 98374, United States	15 September 2020
USA	2005349	WCGIRB – 1019 39th Ave., SE Suite 120, Puyallup, WA 98374, United States	16 September 2020
USA	2005350	WCGIRB – 1019 39th Ave., SE Suite 120, Puyallup, WA 98374, United States	09 September 2020
USA	2005357	WCGIRB – 1019 39th Ave., SE Suite 120, Puyallup, WA 98374, United States	17 August 2020
USA	2005358	WCGIRB – 1019 39th Ave., SE Suite 120, Puyallup, WA 98374, United States	17 August 2020
USA	2005359	WCGIRB – 1019 39th Ave., SE Suite 120, Puyallup, WA 98374, United States	17 August 2020
USA	2005360	WCGIRB – 1019 39th Ave., SE Suite 120, Puyallup, WA 98374, United States	21 August 2020
USA	2005361	WCGIRB – 1019 39th Ave., SE Suite 120, Puyallup, WA 98374, United States	13 August 2020
USA	2005363	WCGIRB – 1019 39th Ave., SE Suite 120, Puyallup, WA 98374, United States	28 August 2020
USA	2005364	WCGIRB – 1019 39th Ave., SE Suite 120, Puyallup, WA 98374, United States	28 August 2020
USA	2005376	WCGIRB – 1019 39th Ave., SE Suite 120, Puyallup, WA 98374, United States	27 August 2020
USA	2005378	WCGIRB – 1019 39th Ave., SE Suite 120, Puyallup, WA 98374, United States	16 September 2020

Country	Centre no.	Name and address of IEC/IRB	Date of Approval
USA	2005379	WCGIRB – 1019 39th Ave., SE Suite 120, Puyallup, WA 98374, United States	09 September 2020
USA	2005385	WCGIRB – 1019 39th Ave., SE Suite 120, Puyallup, WA 98374, United States	06 October 2020
USA	2005421	WCGIRB – 1019 39th Ave., SE Suite 120, Puyallup, WA 98374, United States	13 October 2020

Table S5. Independent ethics committee or institutional review board approvals. IEC, independent ethics committee; IRB, independent review board; WCG, Western Institutional Review Board-Copernicus Group.

Supplementary References

1. Wilkins, D.; Aksyuk, A.A.; Ruzin, A.; Tuffy, K.M.; Green, T.; Greway, R.; Fikes, B.; Bonhomme, C.J.; Esser, M.T.; Kelly, E.J. Validation and performance of a multiplex serology assay to quantify antibody responses following SARS-CoV-2 infection or vaccination. *Clin Transl Immunology*. **2022**, *11*, e1385. https://doi.org/10.1002/cti2.1385.
2. Aksyuk, A.A.; Bansal, H.; Wilkins, D.; Stanley, A.M.; Sproule, S.; Maaske, J.; Sanikommui, S.; Hartman, W.R.; Sobieszczyk, M.E.; Falsey, A.R.; et al. AZD1222-induced nasal antibody responses are shaped by prior SARS-CoV-2 infection and correlate with virologic outcomes in breakthrough infection. *Cell Reports*. **2023**, *4*, 100882. https://doi.org/10.1016/j.xcrm.2022.100882.
3. Falsey, A.R.; Sobieszczyk, M.E.; Hirsch, I.; Sproule, S.; Robb, M.L.; Corey, L.; Neuzil, K.M.; Hahn, W.; Hunt, J.; Mulligan, M.J.; et al. Phase 3 safety and efficacy of AZD1222 (ChAdOx1 nCoV-19) Covid-19 vaccine. *New Engl J Med*. **2021**, *385*, 2348–2360. https://doi.org/10.1056/NEJMoa2105290.
4. Sobieszczyk, M.E.; Maaske, J.; Falsey, A.R.; Sproule, S.; Robb, M.L.; Frenck, R.W., Jr.; Tieu, H.V.; Mayer, K.H.; Corey, L.; Neuzil, K.M.; et al. Durability of protection and immunogenicity of AZD1222 (ChAdOx1 nCoV-19) COVID-19 vaccine over 6 months. *J Clin Invest*. **2022**, *132*, e160565. https://doi.org/10.1172/JCI160565.
